# Evidence of Active-Forgetting Mechanisms? Blocking Arachidonic Acid Release May Slow Forgetting of Sensitization in *Aplysia*

**DOI:** 10.1523/ENEURO.0516-23.2024

**Published:** 2024-04-04

**Authors:** Robert J. Calin-Jageman, Bryan Gonzalez Delgadillo, Elise Gamino, Zayra Juarez, Anna Kurkowski, Nelly Musajeva, Leslie Valdez, Diana Wittrock, Theresa Wilsterman, Jashui Zarate Torres, Irina E. Calin-Jageman

**Affiliations:** Neuroscience Program, Dominican University, River Forest, Illinois 60305

**Keywords:** forgetting, long-term memory, neuromodulation, second messenger signaling

## Abstract

Long-term sensitization in *Aplysia* is accompanied by a persistent up-regulation of mRNA encoding the peptide neurotransmitter Phe-Met-Arg-Phe-amide (FMRFa), a neuromodulator that opposes the expression of sensitization through activation of the arachidonic acid second-messenger pathway. We completed a preregistered test of the hypothesis that FMRFa plays a critical role in the forgetting of sensitization. *Aplysia* received long-term sensitization training and were then given whole-body injections of vehicle (*N *= 27), FMRFa (*N *= 26), or 4-bromophenacylbromide (4-BPB; *N *= 31), a phospholipase inhibitor that prevents the release of arachidonic acid. FMRFa produced no changes in forgetting. 4-BPB decreased forgetting measured 6 d after training [*d*_s _= 0.55 95% CI(0.01, 1.09)], though the estimated effect size is uncertain. Our results provide preliminary evidence that forgetting of sensitization may be a regulated, active process in *Aplysia*, but could also indicate a role for arachidonic acid in stabilizing the induction of sensitization.

## Significance Statement

Forgetting plays an essential role in memory function as both excessive and insufficient forgetting are related to profound disruptions of mental health. Our results provide preliminary evidence that the forgetting of sensitization in *Aplysia* is a regulated process that can be delayed by blocking arachidonic acid production. This work contributes to ongoing efforts to understand the neurobiology of forgetting.

## Introduction

Although long-term memories can last a lifetime, the majority are forgotten, becoming progressively less likely to be recalled (e.g., Bahrick, 1984). Recent evidence suggests that forgetting is an active process, reflecting organized cell and molecular systems that work to oppose the expression of a long-term memory (Davis and Zhong, 2017; Medina, 2018; Ryan and Frankland, 2022). For example, in fruit flies forgetting of an olfactory memory is produced through postlearning activation of a set of dopaminergic neurons; blocking this activity forestalls forgetting and enhancing activation speeds forgetting (Berry et al., 2012; Himmelreich et al., 2017). In addition, the Rho family of G proteins seem to play an evolutionarily conserved role in forgetting, as manipulation of Rac1 bidirectionally regulates forgetting in mice (Liu et al., 2016; O’Leary et al., 2023) and flies (Shuai et al., 2010; Zhang et al., 2018) and Rac2 plays a similar role in *Caenorhabditis elegans* (Bai et al., 2022). Active forgetting mechanisms can be coupled to behavior and thus can contribute not only to spontaneous/passive forgetting but also to interference-based forms of forgetting (Berry et al., 2018; Zhang et al., 2018) and to modulation of forgetting by sleep (Berry et al., 2015).

**Figure 1.**
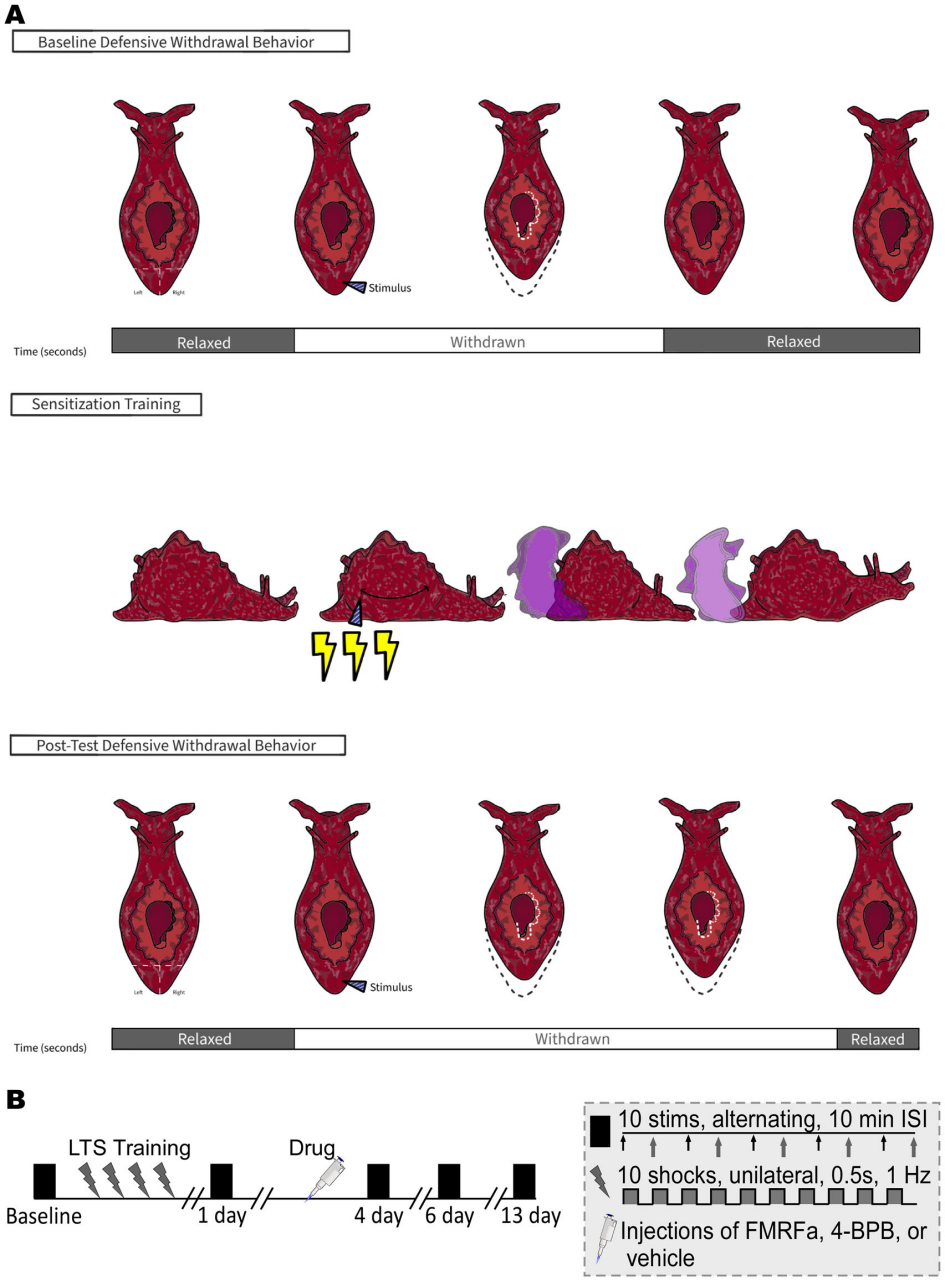
Sensitization in *Aplysia* and experimental protocol. ***A***, Sensitization of the tail-elicited siphon-withdrawal response (T-SWR). The T-SWR (top) is a defensive withdrawal of the siphon elicited by innocuous stimulation to the left or ride side of the tail. This produces a contraction of the siphon, a respiratory structure, lasting several seconds. Sensitization of this reflex (middle) can be produced by applying a noxious stimulus (repeated electrical shock) to one side of the body. This produces pain-related behaviors, including inking (purple cloud) and escape locomotion. It also induces a sensitization memory, expressed as a persistent increase in the duration of the T-SWR on the trained side of the body (bottom). T-SWR duration on the untrained side of the body remains stable (not shown). ***B***, Experimental protocol. T-SWR duration was measured at Baseline and again 1, 4, 6, and 13 d after long-term sensitization training (the 13 d timepoint was exploratory). At each timepoint, five measures were taken on each side of the body at 10 min intervals (solid blocks). Sensitization training consisted of four rounds of strong electrical shock (lightning bolts) applied to one side of the body at 30 min intervals. Drug injections (FMRFa, 4-BPB, or vehicle) were administered after the 1 d posttests (pipette icon; see text for details on dosage and injection schedules).

Recent observations suggest that active-forgetting mechanisms may play a role in sensitization memory in *Aplysia californica*, an important model system for understanding the neurobiology of memory*.* Sensitization is a non-associative form of memory for painful experiences; it is expressed as an increase in responsiveness that persists after exposure to a noxious stimulus (Thompson and Spencer, 1966). In *Aplysia*, long-term sensitization can be induced by repeated strong electrical shocks to one side of the body (Fig. 1*A*; Scholz and Byrne, 1987; Conte et al., 2017). This produces a long-lasting, unilateral increase in the duration of the tail-elicited siphon-withdrawal reflex (T-SWR), a behavior in which an innocuous stimulus to one side of the tail produces a defensive contraction of the siphon, a respiratory structure.

Long-term sensitization is encoded at diverse sites in the neural circuit that mediates the T-SWR (Cleary et al., 1998), but a notable site of plasticity is the ventrocaudal (VC) neuron cluster, a population of nociceptive sensory neurons that innervate the tail and which form excitatory glutamatergic synapses onto tail and siphon motor neurons (Walters et al., 1983). Long-term sensitization training produces a long-lasting increase in VC excitability (Scholz and Byrne, 1987) and long-term facilitation of VC synaptic strength (Frost et al., 1985).

Although *Aplysia* can retain sensitization memories for weeks when given extensive training, our 1 d training protocol produces sensitization that is forgotten within 7–9 d, with T-SWR durations recovering back to pretraining durations (Conte et al., 2017; see Materials and Methods). Forgotten does not mean erased, though, as sensitization can be rapidly reexpressed with a short retraining (Philips et al., 2006; Rosiles et al., 2020).

Long-term sensitization requires changes in gene expression (Sutton et al., 2001), and training produces a complex transcriptional cascade in neurons that contribute to the T-SWR (reviewed in Calin-Jageman et al., 2023). Interestingly, some learning-induced transcriptional changes seem likely to *oppose* sensitization. Specifically, sensitization is accompanied by a robust increase in the mRNA encoding the peptide neurotransmitter Phe-Met-Arg-Phe-amide (FMRFa), with elevated expression observable in the nervous system within 1 d after training and persisting even after sensitization is forgotten (Patel et al., 2018; Perez et al., 2018). In addition, sensitization training increases the expression of a transcript encoding a putative FMRFa GPCR (Conte et al., 2017).

Training-induced increases in transcription related to FMRFa signaling are likely to work against the expression of sensitization memory. FMRFa-releasing neurons inhibit *Aplysia* sensory neurons (Mackey et al., 1987; Small et al., 1992; Xu et al., 1994)*.* Moreover, exogenous application of FMRFa produces long-term depression (Montarolo et al., 1988) and synapse retraction (Schacher and Montarolo, 1991) and prevents physiological changes associated with long-term sensitization (Sweatt et al., 1989; Guan et al., 2002; Fioravante et al., 2006). Thus, increased FMRFa signaling could suppress or even erode sensitization memory, and has been theorized to play a key role in the forgetting of sensitization (Patel et al., 2018).

The effects of FMRFa in *Aplysia* are mediated in part by the arachidonic family of second messengers. FMRFa stimulates the production of arachidonic acid in *Aplysia* sensory neurons, direct application of arachidonic acid mimics most of the inhibitory effects of FMRFa, and blockade of the release of arachidonic acid from cell membranes with the phospholipase inhibitor 4-bromophenacylbromide (4-BPB) prevents FMRFa from inhibiting *Aplysia* sensory neurons (Piomelli et al., 1987b; Critz et al., 1991). FMRFa activates arachidonic acid through activation of GPCR signaling (Volterra and Siegelbaum, 1988).

To test the hypothesis that sensitization is forgotten due to FMRFa signaling we conducted a preregistered experiment in which we manipulated FMRFa signaling after the induction of sensitization and then tracked rates of forgetting. To boost FMRFa signaling, we used repeated direct injection of FMRFa using a dose and timing that produces long-term synaptic depression in cultured *Aplysia* neurons (Fioravante et al., 2008). To block FMRFa signaling, we injected 4-BPB, which blocks the arachidonic acid release that mediates FMRFa signaling (Piomelli et al., 1987b), giving repeated injections across two days to attempt to produce a sustained disruption of any FMRFa contribution to forgetting.

## Materials and Methods

The experimental design is outlined in Figure 1*B*. We followed our preregistered sample-size plan, experimental protocol, and analysis plan precisely, noting three minor deviations below. Preregistration documents and all raw data are posted online: https://osf.io/ny38z/. This manuscript reports how we determined our sample size, all data exclusions, all manipulations, and all measures in the study (Simmons et al., 2012).

### Animals

*A. californica* (75–125 g) were obtained from the RSMAS National Resource for *Aplysia* and maintained at 16°C in one of two 90 gallon aquariums with continuously circulating artificial sea water (Instant Ocean, Aquarium Systems Inc.). *Aplysia* are simultaneous hermaphrodites.

### Long-term sensitization training

A 1 d long-term sensitization training protocol was used (Bonnick et al., 2012), adapted from Wainwright et al. (2002) but with a stronger shock (90 mA vs 60 mA) and a constant-current stimulus. Training consisted of four rounds of noxious shock applied at 30 min intervals to one side of the body with a handheld electrode. Each round of shock consisted of 10 pulses (60 Hz biphasic) of 500 ms duration at a rate of 1 Hz and an amplitude of 90 mA. Our training protocol produces memory that is strongly expressed for several days but which fades in most animals within 1 week (Perez et al., 2018). The side of training was counterbalanced across the manipulation of FMRFa signaling.

### Behavioral measurement

As a behavioral outcome, we measured the duration of the T-SWR (Walters and Erickson, 1986). The reflex was evoked by applying a weak shock to one side of the tail using a handheld stimulator (60 Hz biphasic DC pulse for 500 ms at 2 mA of constant current). T-SWR behavior was measured as the duration of withdrawal from the moment of stimulation to the first sign of siphon relaxation.

To track sensitization memory, we measured T-SWR durations before training (baseline) and then 1, 4, 6, and 13 d after long-term sensitization training (posttests). Measurements were made blind to experimental condition. For each timepoint, behavioral responsiveness was characterized by a series of 10 responses evoked on alternating sides of the body at a 10 min ISI. Scores were split by side of stimulation (trained vs untrained) and averaged (five responses/side for each timepoint characterized).

### Manipulation of FMRFa signaling

After inducing sensitization we manipulated FMRFa signaling by injecting animals with either FMRFa, 4-BPB, or vehicle. Drug manipulations began about 1 h after the 1 d posttests. Drug solutions were created fresh just before administration, first by creating a higher-concentration stock solution and then by diluting and aliquoting individual doses for injection.

For FMRFa, we mimicked a protocol that produces long-term depression in cell culture (Fioravante et al., 2008), administering five injections at 10 min intervals. Each injection was of 0.45 mg of FMRFa dissolved in 1 ml of artificial sea water. This dose was selected because it would produce a final 10 µM concentration in an animal with an internal hemolymph volume of 75 ml.

To impair FMRFa signaling, we injected 4-BPB, which blocks the release of arachidonic acid (Carlson and Levitan, 1990a) and prevents bath application of FMRFa from producing inhibition (Piomelli et al., 1987b). Inspired by findings that chronic NMDA blockers can prevent forgetting (e.g., Sachser et al., 2016), we applied 4-BPB twice daily (1 h intervals) 1 and 2 d after training. This was a fairly limited injection regime, but we did not want to induce carryover effects or illness. Each injection consisted of 0.21 mg 4-BPB dissolved first in 0.1 ml of DMSO (Belkin and Abrams, 1993) and then diluted in 0.9 ml of artificial sea water (1 ml total volume per injection). This dose would produce a final internal concentration of 10 µM, a concentration sufficient to block FMRFa-mediated inhibition in *Aplysia* sensory neurons (Piomelli et al., 1987b).

Animals in the control condition received five injections of vehicle at 10 min intervals 1 d after training, with each injection consisting of 1 ml of either artificial sea water or 10% DMSO in artificial sea water.

Injections were made with 31 gauge syringes and in all conditions produced minimal withdrawal behavior. There were no clear behavioral effects of FMRFa. 4-BPB produced notable but temporary muscle rigidity and two animals died before the 4 d posttests and another two before the 6 d posttests.

### Inclusion criteria

Conducting a fair test of the effect of FMRFa on forgetting requires that all animals exhibit initial sensitization. To that end, we preregistered the criterion that trained animals exhibit at least a 30% increase in T-SWR duration 1 d after training.

### Statistical analysis

Sensitization memory was quantified as the log-fold-change (LFC) in T-SWR duration from baseline: LFC = Log_2_(posttest/baseline). This gives equal weight to increases and decreases in T-SWR duration, and expresses memory strength so that a score of 0 indicates no change from baseline, a score of 1 indicates a doubling of duration, etc. We also report strength of sensitization in an individual group using the standardized mean difference for a single group (Cohen's *d*_1_,), calculated at each posttest timepoint as the mean strength of sensitization divided by the standard deviation. Values of *d*_1_ are reported corrected for bias and with 95% confidence intervals (Lecoutre, 2007; Cousineau and Goulet-Pelletier, 2020).

We planned to measure differences in forgetting by estimating contrasts between the control group and each drug condition at each posttest timepoint. For each set of posttest measures, we report the estimated mean difference in memory strength (LFC_drug_ − LFC_control_) with a 95% confidence interval. We also report the *p* value for a one-sided test of a difference from the control group in the hypothesized direction (more forgetting in the FMRFa group, less forgetting in the 4-BPB group). We also report standardized mean differences between groups (Cohen's *d*_s_), calculated as the mean difference between groups divided by their pooled standard deviation. Values of *d*_s_ are reported corrected for bias and with 95% confidence intervals (Lecoutre, 2007; Cousineau and Goulet-Pelletier, 2020).

### Sample-size determination

We hypothesized that manipulation of FMRFa signaling would produce a large change in the retention of sensitization (*d_s _*≥ 1). We set a sample-size goal of 18 animals per group, sufficient to provide 90% power for our hypothesized effect size for each one-sided test of our planned contrasts between the treated and control groups (α = 0.05).

Our stopping rule was to run shipments of animals (30 per shipment) until achieving 18 qualified animals per condition.

### Deviations from the preregistered protocol

In our original protocol we specified that 4-BPB injections would take place 1, 2, and 3 d after training and that the second round of posttests would take place on day 7. We dropped the third 4-BPB injection based on pilot testing due to concerns there would be carryover effects to behavioral testing on day 4. We also switched the second round of follow-ups to day 6 to minimize the need for weekend testing. Both of these alterations were made prior to primary data collection; we simply failed to update our preregistered plan. Therefore, we do not consider these substantive deviations.

Our original protocol did not specify a 13 d posttest. We extended the protocol to include this timepoint after we observed in the first batch of animals that forgetting was not complete in all groups at the 6 d posttests. The 13 d data presented below are exploratory.

## Results

### Planned analysis: induction of long-term sensitization

**Figure 2.**
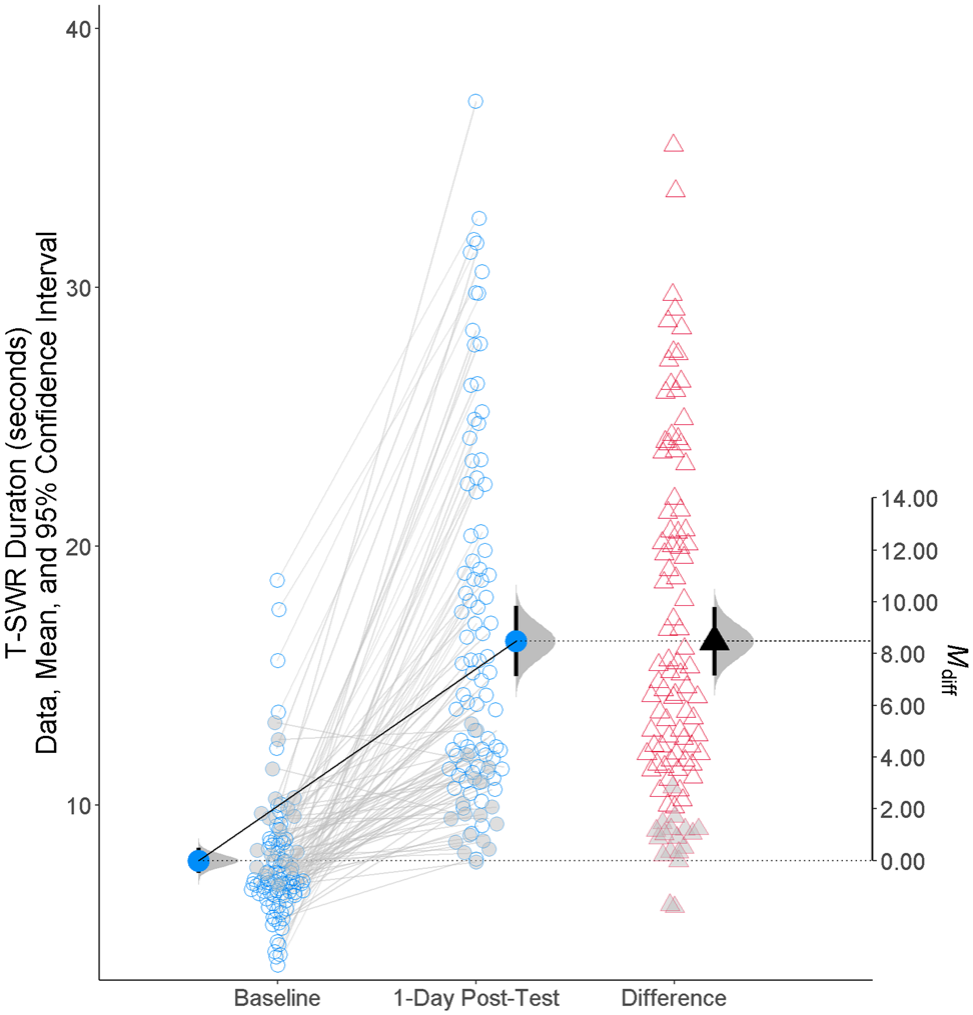
Training produces a long-term sensitization memory. This plot shows the duration of the tail-elicited siphon-withdrawal reflex (T-SWR) before (baseline) and after (1 d posttest) sensitization training. Raw data are presented for each animal (open circles) along with their change in T-SWR duration (open triangles). The closed shapes with error bars represent mean responses with 95% confidence intervals. The animals shown with gray are those that did not meet the prespecified criterion for exhibiting a strong sensitization memory (at least a 30% increase in T-SWR duration). These animals were excluded from the experiment; the remainder was then assigned to a condition for manipulating FMRFa signaling.

After baseline measures, all animals received long-term sensitization training (*N *= 102). As expected, training produced robust long-term sensitization memory, with mean T-SWR responses increasing from an average of 7.8 s at baseline to 16.3 s when observed 1 d after training, a 2-fold increase [Fig. 2; LFC = 1.00 95% CI(0.87, 1.13), *d*_1 _= 1.49 95% CI (1.21, 1.77), Table 1].

**Table 1.** Statistical table

Data structure	Type of estimate	Estimate
All animals (*N *= 102), log-fold change in T-SWR duration on the trained side from baseline to 1 d posttest	Mean log-fold change (LFC), where 0 indicates no change in behavior and values above 0 indicated sensitization *d*_1_ (standardized mean difference for a single group against a reference value of 0)	LFC = 1.00 95% CI[0.87, 1.13] *d*_1 _= 1.49 95% CI [1.21, 1.77]
Animals that passed the learning criteria (*N *= 84), log-fold change in T-SWR duration on the trained side from baseline to 1 d posttest	Mean log-fold change (LFC) *d*_1_ (standardized mean difference for a single group against a reference value of 0)	LFC = 1.19 95% CI[1.06, 1.31] *d*_1 _= 2.07 95% CI[1.69, 2.45]
Animals that passed the learning criteria (*N *= 84), log-fold change in T-SWR duration on the untrained side from baseline to 1 d posttest	Mean log-fold change (LFC) *d*_1_ (standardized mean difference for a single group against a reference value of 0)	LFC = −0.10 95% CI [−0.19, 0.00] *d*_1 _= −0.22 95% CI[−0.44, 0.00]
Control group only, log-fold change in T-SWR duration on the trained side from baseline to 4 d posttest	Mean log-fold change (LFC) *d*_1_ (standardized mean difference for a single group against a reference value of 0)	LFC = 0.80 95% CI[0.57, 1.02] *d*_1 _= 1.28 95% CI[0.76, 1.79]
Control group only, log-fold change in T-SWR duration on the trained side from baseline to 6 d posttest	Mean log-fold change (LFC) *d*_1_ (standardized mean difference for a single group against a reference value of 0)	LFC = 0.44 95% CI[0.19, 0.69] *d*_1 _= 0.66 95% CI[0.24, 1.08]
Planned contrast between control group and FMRFa group, comparing strength of sensitization (trained side) at 4 d posttest	Mean difference between groups *d*_s_ (standardized mean difference between groups) One-sided test of significantly less FMRFa memory strength than controls against a null of no reduction, α = 0.05	LFC_FMFRa_ − LFC_control _= 0.04 95% CI[−0.27, 0.36] *d*_s _= 0.07 95% CI[−0.46, 0.61] Fail to reject; wrong direction
Planned contrast between control group and FMRFa group, comparing strength of sensitization (trained side) at 6 d posttest	Mean difference between groups *d*_s_ (standardized mean difference between groups) One-sided test of significantly less FMRFa memory strength than controls against a null of no reduction, α = 0.05	LFC_FMFRa_ − LFC_control _= 0.10 95% CI[−0.26, 0.46] *d*_s _= 0.10 95% CI[−0.39, 0.69] Fail to reject; wrong direction
Planned contrast between control group and 4-BPB group, comparing strength of sensitization (trained side) at 4 d posttest	Mean difference between groups *d*_s_ (standardized mean difference between groups) One-sided test of significantly more 4-BPB memory strength than controls against a null of no reduction, α = 0.05	LFC_4-BPB_ − LFC_control _= 0.09 95% CI[−0.22, 0.40] *d*_s _= 0.15 95% CI[−0.37, 0.68] Fail to reject the null; *p *= 0.28
Planned contrast between control group and 4-BPB group, comparing strength of sensitization (trained side) at 6 d posttest	Mean difference between groups *d*_s_ (standardized mean difference between groups) One-sided test of significantly more 4-BPB memory strength than controls against a null of no reduction, α = 0.05	LFC_4-BPB_ − LFC_control _= 0.36 95% CI[0.01, 0.72] *d*_s _= 0.55 95% CI[0.01, 1.09] *p *= 0.02
Planned contrast between control group and 4-BPB group, comparing changes in T-SWR duration on the untrained side at 4 d posttest	Mean difference between groups *d*_s_ (standardized mean difference between groups)	LFC_4-BPB_ − LFC_control _= −0.19 95% CI[−0.56, 0.17] *d*_s _= −0.28 95% CI[−0.80, 0.25]
Planned contrast between control group and 4-BPB group, comparing changes in T-SWR duration on the untrained side at 6 d posttest	Mean difference between groups *d*_s_ (standardized mean difference between groups)	LFC_4-BPB_ − LFC_control _= −0.03 95% CI[−0.40, 0.34] *d*_s _= −0.04 95% CI[−0.58, 0.50]
Exploratory analysis: 4-BPB group only, log-fold change in T-SWR duration on the trained side from baseline to 13 d posttest	Mean log-fold change (LFC)	LFC = 0.34 95% CI[0.07, 0.61]
Exploratory analysis: contrast between control group and 4-BPB group, comparing strength of sensitization (trained side) at 13 d posttest	Mean difference between groups *d*_s_ (standardized mean difference between groups) One-sided test of significantly more 4-BPB memory strength than controls against a null of no reduction, α = 0.05	LFC_4-BPB_ − LFC_control _= 0.19 95% CI[−0.18, 0.56] *d*_s _= 0.29 95% CI[−0.26, 0.84] Fail to reject the null; *p *= 0.15

Although this was a strong average level of memory expression, 18 animals did not meet our preregistered criterion of at least a 30% increase in T-SWR duration on the side of training. These animals were excluded from the experiment. This left 84 animals exhibiting very strong and consistent sensitization memory [LFC = 1.19 95% CI(1.06, 1.31), *d*_1 _= 2.07 95% CI(1.69, 2.45)]. As expected, the expression of sensitization memory was unilateral, with only modest habituation evident on the untrained side of the body [LFC = −0.10 95% CI (−0.19, 0.00), *d*_1 _= −0.22 95% CI(−0.44, 0.00)].

### Planned analysis: effect of FMRFa manipulation on forgetting

After confirming the expression of sensitization, we manipulated FMRFa signaling by injecting animals with either FMRFa (*N *= 26), 4-BPB (*N *= 31), or vehicle (*N *= 27).

**Figure 3.**
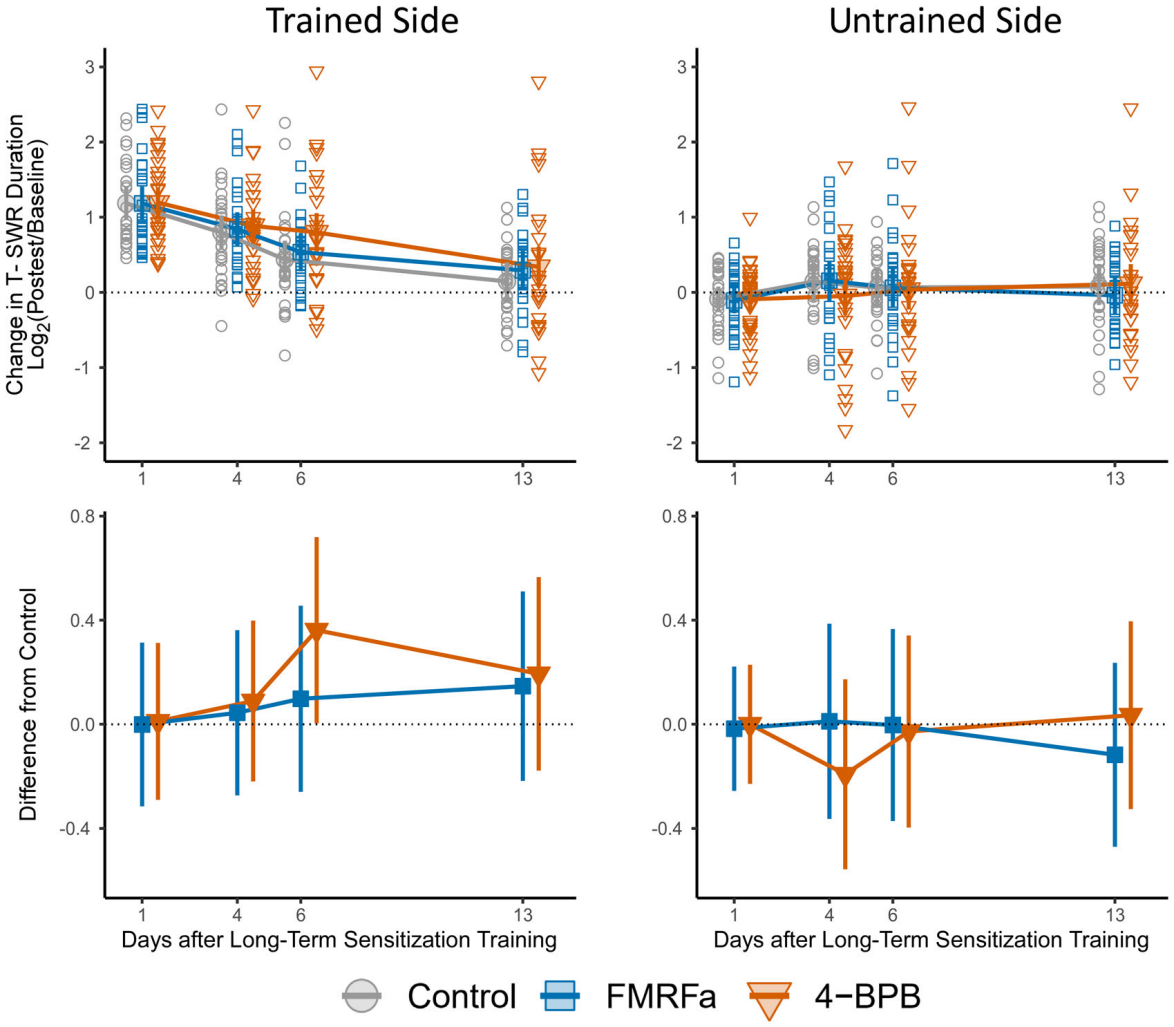
Effects of FMRFa and 4-BPB injections on the forgetting of sensitization. At top are changes in T-SWR duration (Log_2_(posttest/baseline)) after sensitization training in the control (gray circles; *N *= 27), FMRFa (blue squares; *N* *= 26*) and 4-BPB groups (orange triangles; *N = 31*) on the trained (left) and untrained (right) side. Empty symbols show raw scores for each animal; solid symbols connected by lines represent group means; error bars represent 95% confidence intervals. At bottom are estimated mean differences in forgetting when comparing the FMRFa and 4-BPB groups to the Control group. Error bars represent 95% confidence intervals for each difference in means.

We tested our hypothesis of altered forgetting by measuring T-SWR durations 4 and 6 d after training (Fig. 3). In the control group, sensitization memory was still very strong 4 d after training [LFC = 0.80 95% CI(0.57, 1.02), *d*_1 _= 1.28 95% CI(0.76, 1.79)] but then declined to moderate expression 6 d after training, indicating substantial forgetting [LFC = 0.44 95% CI(0.19, 0.69), *d*_1 _= 0.66 95% CI(0.24, 1.08)].

FMRFa injections produced little-to-no change in the time-course of forgetting, with effectively no difference in strength of sensitization at day 4 [LFC_FMFRa_ − LFC_control _= 0.04 95% CI(−0.27, 0.36); *d*_s _= 0.07 95% CI(−0.46, 0.61), one-sided hypothesis test: wrong direction] and day 6 [LFC_FMFRa_ − LFC_control _= 0.10 95% CI(−0.26, 0.46); *d*_s _= 0.10 95% CI(−0.39, 0.69), one-sided hypothesis test: wrong direction].

For animals injected with 4-BPB there was little difference from controls at day 4, when little forgetting had occurred [LFC_4-BPB_ − LFC_control _= 0.09 95% CI(−0.22, 0.40); *d*_s _= 0.15 95% CI(−0.37, 0.68), *p *= 0.28], but at day 6, the animals injected with 4-BPB showed stronger retention of sensitization [LFC_4-BPB_ − LFC_control _= 0.36 95% CI(0.01, 0.72); *d*_s_ = 0.55 95% CI(0.01, 1.09), *p *= 0.02], though note that the confidence interval is long and cannot rule out effects close to 0. This difference in forgetting is probably not due to non-specific effects of 4-BPB on the T-SWR reflex, as responses on the untrained side were stable and remained very close to those observed in controls. This was true both at day 4 [LFC_4-BPB_ − LFC_control _= −0.19 95% CI(−0.56, 0.17); *d*_s _= −0.28 95% CI(−0.80, 0.25)] and at day 6 [LFC_4-BPB_ − LFC_control _= −0.03 95% CI(−0.40, 0.34); *d*_s _= −0.04 95% CI(−0.58, 0.50)].

### Exploratory analysis: long-term effect of FMRFa manipulation on forgetting

Because forgetting was not complete in all groups by 6 d, we added a 13 d posttest; analysis of this data is exploratory. At 13 d, animals in the 4-BPB group still expressed weak but clear sensitization memory [LFC = 0.34 95% CI(0.07, 0.61)] while the control animals showed only a slight elevation in T-SWR scores that was compatible with population effects of no remaining sensitization [LFC = 0.15 95% CI(−0.11, 0.40)]. The difference between these groups was compatible with a continued effect of 4-BPB but also with no change in forgetting [LFC_4-BPB_ − LFC_control _= 0.19 95% CI(−0.18, 0.56); *d*_s_ = 0.29 95% CI(−0.26, 0.84), *p *= 0.15].

## Discussion

Our results suggest that blocking arachidonic acid release with 4-BPB can slow the forgetting of sensitization in *Aplysia*, though we cannot rule out effect sizes that would be too small to regularly detect with achievable sample sizes. This finding provides qualified support for the idea that forgetting of sensitization is an active process and documents another phylum in the animal kingdom (Mollusca) in which forgetting can be manipulated through alterations in second-messenger signaling (Liu et al., 2016; Zhang et al., 2018; Bai et al., 2022; O’Leary et al., 2023). Definitive evidence for active forgetting of sensitization will require replication and examining if enhancing arachidonic acid release can speed forgetting.

An alternative interpretation of these results is that 4-BPB interferes not with forgetting but with the stabilization of sensitization memory induction. That is, even though our manipulations were applied 1 d after training, it may be that induction mechanisms are still fragile at this timepoint. For example, maintenance of the cellular correlates of sensitization can be disrupted when CPEB is depleted 1–2 d after induction but not at 3 d after induction, suggesting a time-window during which induction mechanisms become stabilized (Miniaci et al., 2008). Investigating additional timepoints might be able to arbitrate between these interpretations, but it may also be that there is not a mechanistic difference between active forgetting and the destabilization of induction mechanisms.

This experiment did not support our hypothesis that FMRFa is the key signal regulating forgetting of sensitization. First, we did not observe an enhancement of forgetting with FMRFa injection despite a sample-size planned to provide high power to detect a large effect. Second, although we did observe the hypothesized effect of 4-BPB, this could be due to other signaling pathways that activate arachidonic acid (Piomelli et al., 1987a). Although not supported, our hypothesis is not yet refuted. First, although FMRFa application is impactful in neurons cultured in hemolymph, we cannot be certain that the dose we applied via whole-animal injection had good bioavailability in the nervous system. In addition, the dose we used is impactful in cultured neurons that lack any FMRFa inputs, so we also cannot rule out saturation effects in our whole-animal protocol, especially given that sensitization causes FMRFa release (Mackey et al., 1987). An additional test with stronger impact may be warranted given the other lines of supportive evidence: the clear increase in FMRFa transcription that occurs with sensitization (Conte et al., 2017), the ability of FMRFa to depress synapses that contribute to the T-SWR (Montarolo et al., 1988), and the fact that FMRFa triggers the release of arachidonic acid (Piomelli et al., 1987b), which does seems to regulate aspects of forgetting. We are now planning an additional test of the role of FMRFa in forgetting using semi-intact preparations in which FMRFa, 4-BPB, and arachidonic acid can be applied directly to the nervous system. This would also make it possible to better validate the impacts of these manipulations.

In vertebrates, arachidonic acid has been linked to memory enhancement rather than forgetting. Specifically, supplementing rat diets with arachidonic acid has been reported to protect against anesthesia-induced memory deficits (Li et al., 2015) and restores age-related declines in spatial memory (Okaichi et al., 2006, 2005) and LTP maintenance (McGahon et al., 1997). In contrast, we observed that blocking arachidonic acid release with 4-BPB may slow forgetting, suggesting that arachidonic acid functions in *Aplysia* to reduce the expression of sensitization memory. This is consistent with previous investigations of the neuronal effects of arachidonic acid in *Aplysia*. Specifically, direct application of arachidonic acid to cultured *Aplysia* neurons produces long-term depression of sensory-to-motor synapses and retraction of sensory-neuron synaptic processes (Schacher et al., 1993). These synaptic effects are the opposite of those induced by long-term sensitization training (Frost et al., 1985; Wainwright et al., 2002).

Treatment with 4-BPB produced clear changes in T-SWR behavior only on the side of training. This suggests that sensitization training produces an increase in arachidonic acid signaling that confers a susceptibility to 4-BPB treatment. Consistent with this hypothesis, strong neuronal activity (which sensitization training produces) increases the production of arachidonic acid in *Aplysia* neurons (Piomelli et al., 1987b; Carlson and Levitan, 1990b). Integrating our current results with these previous findings, we propose that sensitization training produces not only the synaptic and cellular changes that help encode sensitization memory, but also an increase in arachidonic acid signaling that helps eventually erode that memory, producing forgetting. According to this model, learning triggers not only the processes needed to encode a memory but also signaling pathways that will eventually weaken expression of the memory. At this stage, though, our results remain primarily suggestive; additional study will be needed to better resolve the roles of FMRFa and arachidonic acid in the forgetting of sensitization and to disentangle potential effects on forgetting from delayed impacts on induction.

## Synthesis

Reviewing Editor: Christine Portfors, Washington State University Decisions are customarily a result of the Reviewing Editor and the peer reviewers coming together and discussing their recommendations until a consensus is reached. When revisions are invited, a fact-based synthesis statement explaining their decision and outlining what is needed to prepare a revision will be listed below. The following reviewer(s) agreed to reveal their identity: Wayne Sossin, Xin Deng.

The reviewers are in agreement that the manuscript is a valuable addition to the literature and provides support for using Aplysia as a feasible model system for studying the neurobiology of forgetting at the single cell and circuit level. The manuscript would be improved with clarifications in methodology and better descriptions of how aspects of the methodology (as outlined below) may impact the interpretation for the results.

Specific comments:

The treatments with 4-BPB were fundamentally difference than the treatments with FMRFamide in a number of ways and these differences in treatment were not considered in the discussion.

- 4-BPB was injected both after day 1 and day 2, while FMRFamide was administered only after day 1. This was also different from the preregistered plan where 4-BPB was proposed to be injected on days 1, 2 and 3. The reasoning for the difference in application of the two drugs was not explicitly stated, but there are waves of changes underlying long-term memory formation and how forgetting is induced may be different depending on the day examined.

- Since FMRFamide is endogenously produced by the animal, it is not clear that its levels are not already saturated by the increase in mRNA observed after sensitization.

- Indeed in 4-BPB is acting by blocking endogenous FMRFamide signaling this suggests that this signaling is already present in the absence of added FMRFamide. Cultured neurons lack an endogenous source of FMRFamide, so in this case it would have to be added exogenously to the culture.

The discussion is completely centered on forgetting, but enhancement of threshold memory processes can prolong memory in the absence of inhibition of forgetting. Perhaps this is semantic, but it seems different and it is not clear how this experiment distinguishes between these two concepts. As a simple example, if inhibitors of CREB are repressed, memory from a single stimulus lasts much longer, but it is not thought to be due to an increase in forgetting but the lowering of a threshold for memory formation. Since it is not clear how long after the initial stimulus the cellular memories are actually formed, decreasing thresholds for long-term memory formation days after learning may be a mechanism of prolonging the life time of memory .

The authors suggest that FMRFa plays critical role in regulating forgetting of LTS by triggering the release of arachidonic acid, which prevents expression of LTS/promote forgetting. To manipulate FMRFa signaling, the authors injected FMRFa and 4-BPB to increase and decrease arachidonic acid level, respectively. However, the manuscript lacks the evidence that how arachidonic acid level was changed by FMRFa and 4-BPB. Validation of the changes of arachidonic acid level in the three experimental groups during forgetting might be needed to illustrate involvement of arachidonic acid in forgetting of sensitization.

The authors planned to systemically inject FMRFa into the animals at 1 day post LTS training to increase FMRFa signaling. The manuscript lacks sufficient rationality of applying this protocol. Some points need to be clarified: 1) The FMRFa's resistance to hemolymph peptidases/proteases; 2) FMRFa is already released during LTS training (Mackey et al., 1987; Guan et al., 2002; Fioravante et al., 2008), will injecting the peptide after LTS still increase the FMRFa signaling?

The administration of FMRFa and 4-BPB were different: FMRFa was injected 5 times in 1 day, while 4-BPB was injected 2 times each day for two days. The manuscript lacks the explanation for this difference. According to the results, only the hypothesized effect of 4-BPB was observed. The reason that FMRFa produced little change in forgetting might be that the method for administration of FMRFa cannot be effective for 5 days, when LTS started to be forgotten.

Minor comments:

1. The LTS training protocol applied in the study is different to the previous protocols cited in the introduction (e.g., Scholz and Bryne, 1987; Cleary et al., 1998). The LTS protocol used by previous ones induced sensory neuron increased excitability and synaptic plasticity (e.g., Scholz and Bryne, 1987; Cleary et al., 1998), but will not last for many days (Wainwright et al., 2002). The authors' LTS protocol is more extensive that last for at least 4 days and induced synaptic plasticity without changing sensory neuron excitability (Wainwright et al., 2002). This discrepancy should be pointed out.

2. Page 3 Line 41, please provide full name for the abbreviation "VC".

3. Page 5 Line 90, 1 Hz instead of "1hz".

4. Page 5 Line 94, full name of T-SWR has been expanded in the introduction.

5. Page 5 Line 96, 2 mA instead of "2ma".

6. Page 6 Line 112, lack a reference for the statement.

7. Page 6 Line 123, 31-"gauge".

8. Page 9 Line 177, figure 3 instead of "figure 2".

## Author Response

The treatments with 4-BPB were fundamentally difference than the treatments with FMRFamide in a number of ways and these differences in treatment were not considered in the discussion.

- 4-BPB was injected both after day 1 and day 2, while FMRFamide was administered only after day 1. This was also different from the preregistered plan where 4-BPB was proposed to be injected on days 1, 2 and 3. The reasoning for the difference in application of the two drugs was not explicitly stated, but there are waves of changes underlying long-term memory formation and how forgetting is induced may be different depending on the day examined.

- Since FMRFamide is endogenously produced by the animal, it is not clear that its levels are not already saturated by the increase in mRNA observed after sensitization.

- Indeed in 4-BPB is acting by blocking endogenous FMRFamide signaling this suggests that this signaling is already present in the absence of added FMRFamide. Cultured neurons lack an endogenous source of FMRFamide, so in this case it would have to be added exogenously to the culture.

First - thank you for catching the protocol discrepancy. We had, indeed, specified a 3rd round of 4-BPB injections in our original pre-registration. During pilot testing we became concerned about carryover effects into the behavioral testing on day 4 and dropped the 3rd round from the protocol to ensure full time for drug clearance between injection and behavioral testing. We had not documented this deviation in the manuscript, but have now updated appropriately.

In addition, we have updated the discussion to raise the issue of saturation and the fact that our protocol was based on cultured neurons that lack FMRFa inputs, both strong points we had not previously considered.

The discussion is completely centered on forgetting, but enhancement of threshold memory processes can prolong memory in the absence of inhibition of forgetting. Perhaps this is semantic, but it seems different and it is not clear how this experiment distinguishes between these two concepts. As a simple example, if inhibitors of CREB are repressed, memory from a single stimulus lasts much longer, but it is not thought to be due to an increase in forgetting but the lowering of a threshold for memory formation. Since it is not clear how long after the initial stimulus the cellular memories are actually formed, decreasing thresholds for long-term memory formation days after learning may be a mechanism of prolonging the life time of memory .

This is a good point: we can't *completely* dissociate a disruption of forgetting mechanisms from an enhancement of memory formation mechanisms. In our case, manipulation did not occur until a bit more than 1 day after training, a time point when memory has already been formed and is strongly expressed. Therefore, temporally, this suggests more of an impact on memory maintenance than on memory induction and/or threshold. It is not clear, however, how long induction mechanisms take to stabilize, and indeed manipulations that alter CPEB function can disrupt memory expression for up to a few days after induction but not longer, and one interpretation of that finding is that there is a fairly extended window during which memory induction mechanisms fully stabilize. Testing for a time-based sensitivity to 4-BPB might help resolve between these interpretations, but as you point out these might be different labels for the same underlying mechanism. This is an important set of points, and we have now expanded the discussion to raise these issues.

The authors suggest that FMRFa plays critical role in regulating forgetting of LTS by triggering the release of arachidonic acid, which prevents expression of LTS/promote forgetting. To manipulate FMRFa signaling, the authors injected FMRFa and 4-BPB to increase and decrease arachidonic acid level, respectively. However, the manuscript lacks the evidence that how arachidonic acid level was changed by FMRFa and 4-BPB. Validation of the changes of arachidonic acid level in the three experimental groups during forgetting might be needed to illustrate involvement of arachidonic acid in forgetting of sensitization.

It's true; there is always going to be some ambiguity about drug effects. Although we did not measure arachidonic-acid level changes ourselves, the pathways we have stipulated are well-established (Critz et al., 1991; Piomelli et al, 1987; Schacher et al., 1993): • FMRFa application increases the release of arachidonic acid in Aplysia. • Direct application of arachidonic acid mimics the effects of FMRFa, including not only its direct inhibitory effects but also its ability to induce long-term synaptic depression. • 4-BPB is a well-validated blocker of arachindonic acid release, and applying 4-BPB to Aplysia ganglia prevents synaptic effects of FMRFa.

We have updated the introduction and discussion to emphasize these prior findings, but also to acknowledge the intrinsic ambiguity of interpretating the effects we observed.

The authors planned to systemically inject FMRFa into the animals at 1 day post LTS training to increase FMRFa signaling. The manuscript lacks sufficient rationality of applying this protocol. Some points need to be clarified: 1) The FMRFa's resistance to hemolymph peptidases/proteases; 2) FMRFa is already released during LTS training (Mackey et al., 1987; Guan et al., 2002; Fioravante et al., 2008), will injecting the peptide after LTS still increase the FMRFa signaling? These are all good points, and are related to the overall theme across all reviewer comments to better explain, justify, and interpret the drug applications.

It's true that we adopted a protocol applied in culture for whole-animals, and that this raises some issues: both that there could be more degradation/penetration issues in whole animals (though cultured neurons are grown in a hymolymph-based solution) as well as saturation (because cultured neurons are not grown with FMRFa inputs and because sensitization is expected to release and then further boost FMRFa signaling). The issue of saturation is especially important, and not one that we had previously considered (thanks!) We have updated the introduction to clarify the protocol, we have updated the methods section to make more clear how it was adopted from the previous literature, and most importantly we have revised the discussion to make more clear these difficult issues of interpretation.

The administration of FMRFa and 4-BPB were different: FMRFa was injected 5 times in 1 day, while 4-BPB was injected 2 times each day for two days. The manuscript lacks the explanation for this difference. According to the results, only the hypothesized effect of 4-BPB was observed. The reason that FMRFa produced little change in forgetting might be that the method for administration of FMRFa cannot be effective for 5 days, when LTS started to be forgotten.

This comment is part of a consistent theme indicating that we did not do enough in the manuscript to explain, justify, and interpret the primary manipulation. We have attempted to thoroughly address this and the related comments through revisions throughout the paper.

Minor comments:

1. The LTS training protocol applied in the study is different to the previous protocols cited in the introduction (e.g., Scholz and Bryne, 1987; Cleary et al., 1998). The LTS protocol used by previous ones induced sensory neuron increased excitability and synaptic plasticity (e.g., Scholz and Bryne, 1987; Cleary et al., 1998), but will not last for many days (Wainwright et al., 2002). The authors' LTS protocol is more extensive that last for at least 4 days and induced synaptic plasticity without changing sensory neuron excitability (Wainwright et al., 2002). This discrepancy should be pointed out.

This is correct: We use an enhanced version of the original Scholz & Byrne protocol (Bonnick et al., 2012; Conte et al., 2017). In our lab, we use a 90 mA shock (compared to Scholz & Byrne's 60 mA shock) with a constant current stimulus (compared to Scholz & Byrne's AC stimulus with voltage adjusted using a dummy-load to achieve a desired maximum current level). Our adjusted protocol produces stronger changes in gene expression (Bonnick et al. 2012) and longer-lasting sensitization (Conte et al., 2017): it is strongly expressed for at least 4 days and fades in most animals within 7-9 days.

We have now updated the methods and introduction to clarify these distinctions.

2. Page 3 Line 41, please provide full name for the abbreviation "VC".

Fixed

3. Page 5 Line 90, 1 Hz instead of "1hz".

Fixed

4. Page 5 Line 94, full name of T-SWR has been expanded in the introduction.

Fixed

5. Page 5 Line 96, 2 mA instead of "2ma".

Fixed

6. Page 6 Line 112, lack a reference for the statement.

Considered

7. Page 6 Line 123, 31-"gauge".

Fixed

8. Page 9 Line 177, figure 3 instead of "figure 2".

Fixed
